# Risk assessment and disease burden of extreme precipitation on hospitalizations for acute aortic dissection in a subtropical coastal Chinese city

**DOI:** 10.3389/fpubh.2023.1216847

**Published:** 2023-06-29

**Authors:** Yanhu Ji, Jianping Xiong, Zhongjia Yuan, Zepeng Huang, Liping Li

**Affiliations:** ^1^School of Public Health, Shantou University, Shantou, China; ^2^The First Affiliated Hospital of Shantou University Medical College, Shantou, China; ^3^Shantou Central Hospital, Shantou, China; ^4^The Second Affiliated Hospital of Shantou University Medical College, Shantou, China; ^5^Injury Prevention Research Center, Shantou University Medical College, Shantou, China

**Keywords:** acute aortic dissection, disease burden, time series, extreme precipitation, distributed lag nonlinear model

## Abstract

**Background:**

Extreme precipitation events are becoming more frequent due to climate change. The present study aimed to explore the impacts of extreme precipitation on hospitalizations for acute aortic dissection (AAD) and to identify susceptible populations and quantify the corresponding disease burden.

**Methods:**

The present study used a distributed lag nonlinear model (DLNM) with a quasi-Poisson function to investigate the association between extreme precipitation (≥95th percentile) and the risk of hospitalizations for AAD from 2015 to 2020 in Shantou, Guangdong Province, China.

**Results:**

The significant adverse effects of extreme precipitation (relative to no precipitation) on daily AAD hospitalizations lasted from lag 5 [relative risk (RR): 1.0318, 95% confidence interval (CI): 1.0067–1.0575] to lag 9 (RR: 1.0297, 95% CI: 1.0045–1.0555) and reached its maximum at lag 7 (RR: 1.0382, 95% CI: 1.0105–1.0665). Males and older adult individuals (≥60 years) were more susceptible to extreme precipitation. A total of 3.68% (118 cases) of AAD hospitalizations were due to extreme precipitation.

**Conclusion:**

Extreme precipitation was significantly correlated with AAD hospitalizations. Government departments should actively implement extreme precipitation intervention measures to strengthen the protection of males and the older adult (≥60 years) and effectively reduce AAD hospitalizations.

## Introduction

Acute aortic dissection (AAD) is a critical cardiovascular condition that is characterized by tearing of the intimal layer of the aorta wall, with blood entering the middle layer through the intimal tear and then separating along the aorta axis to form true and false lumens (1). AAD has a rapid onset, rapid progression, high complication rate and high fatality rate (2). Population-based studies indicate that the incidence of AAD is 2.6 to 3.5 cases per 100,000 person-years (3), with an obvious upwards trend in recent years. Mortality rates from AAD are as high as 90% in the absence of intervention, with most deaths occurring within 48 h of AAD onset (4). Therefore, the identification of potential risk factors for AAD is critical to addressing this public health issue. Common causes of AAD include uncontrolled hypertension, arteriosclerosis, and hereditary connective tissue diseases (5). Air pollutants are also associated with the incidence of AAD (6, 7).

With the intensification of global climate change, extreme meteorological events (e.g., drought, heat wave, cold wave, and extreme precipitation) continue to occur, and their impact on human health has attracted great attention. A large number of studies have investigated the effects of extreme meteorological factors and acute cardiovascular events, such as AAD, but existing studies have primarily focused on temperature (8–12). There are few studies on the association of other meteorological factors, particularly rainfall, with AAD, and the conclusions are inconsistent. For example, studies in three Chinese cities, Beijing (13), Hong Kong (14), and Urumqi (15), showed that the incidence of AAD was not associated with daily rainfall, but a study in western Japan suggested that rainy days were a risk factor for cardiovascular events in older adults (16). Notably, no studies have examined the risk of extreme precipitation and the incidence of AAD.

Extreme precipitation events have increased across China in recent years, especially in the coastal areas of southern China (17–19). Therefore, the present study was performed in Shantou city, southern China, with the following three objectives: first, to identify the association between extreme precipitation and AAD hospitalizations in Shantou city; second, to identify vulnerable populations with extreme precipitation; and finally, to quantify the burden of AAD hospitalizations due to extreme precipitation. The present study supplements the current limited evidence on the association between extreme precipitation and AAD and provides a reference for relevant authorities to formulate active and effective preventive measures.

## Materials and methods

### Study area

**Figure 1:**
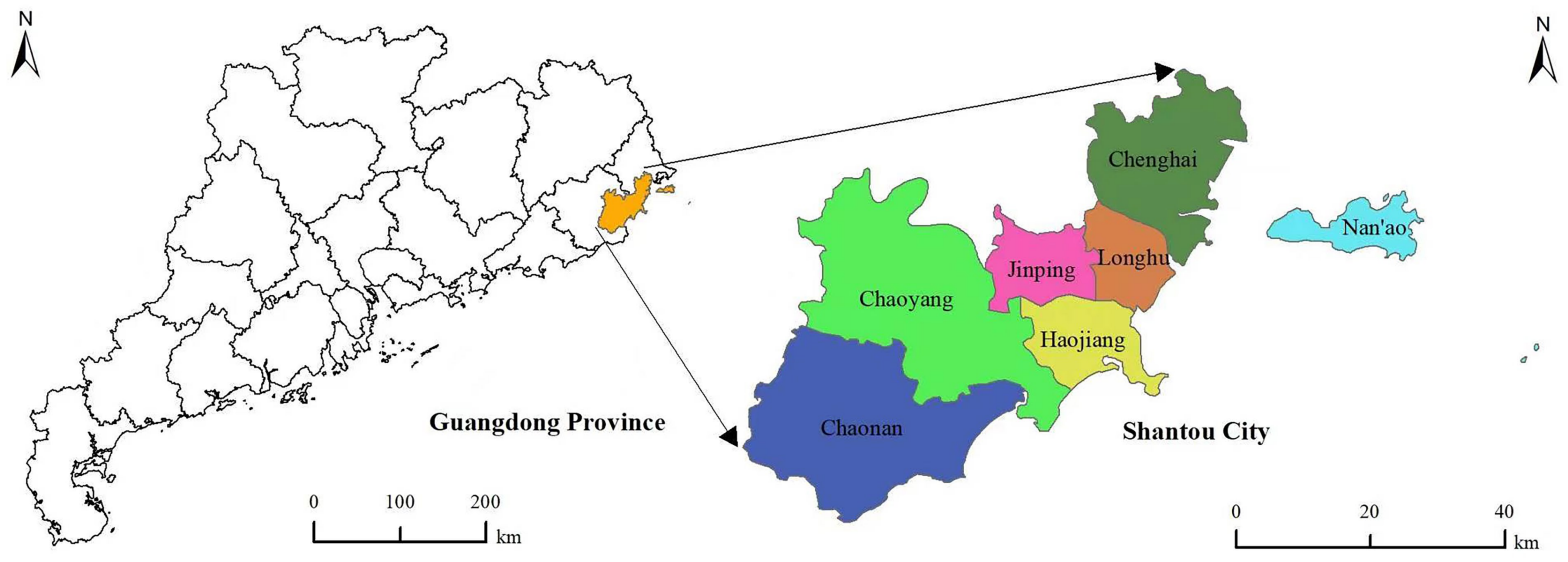
Geographical location of Shantou, China.

Shantou is located in southeastern China, and it is the only city in mainland China with an inner bay. Shantou had a total area of 2,199 square kilometres, a permanent population of 5,530,400, and a population density of 2,515 people per square kilometre in 2021, and it ranks 7th among all cities in China. Shantou city has a subtropical monsoon climate that is characterized by winters without severe cold and summers without severe heat. The location of Shantou city in China is shown in Figure 1.

### Data collection

The information of daily patients with AAD from January 1, 2015 to December 31, 2020 was obtained from four tertiary general hospitals in Shantou: the First Affiliated Hospital of Shantou University Medical College, Shantou Chaonan Minsheng Hospital, the Second Affiliated Hospital of Shantou University Medical College and Shantou Central Hospital. These four general hospitals have high medical levels and advanced medical equipment, and these four hospitals are the first choice for AAD patients seeking treatment. AAD cases were identified according to the International Classification of Diseases (ICD10 code: I71.0), and patient information primarily included date of hospitalization, sex, age and residential address. Only cases with home addresses in Shantou city were included in this study. Meteorological factors and air pollution data for the same period were obtained from the China Meteorological Science Data Sharing Service Network^1^ and the local Environmental Protection Authority, respectively.

Due to the different geographical characteristics and seasonal precipitation distributions, there is no clear and unified definition of extreme precipitation. Consistent with several previously published studies in China, we also used the percentile approach to define extreme precipitation in the present study (20–23). We used the 95th percentile as the cut-off point to divide the daily precipitation into three ordinal categories: (1) equal to 0 mm precipitation (reference, no precipitation); (2) 0–95 percentile precipitation (moderate precipitation); and (3) ≥95th percentile precipitation (extreme precipitation).

### Statistical analysis

Daily hospitalizations for AAD approximately followed an overdispersion distribution. Therefore, the present study used a generalized linear model (quasi-Poisson function) combined with DLNM to investigate the association between extreme precipitation and AAD hospitalizations (24). Supplementary Table A1 shows the Spearman correlation coefficients between environmental variables in Shantou, China. We found a high negative correlation between mean temperature and atmospheric pressure (*r* = −0.86, *p* < 0.001), so the two factors were not included in the same model. Other variables had lower correlations with each other. The potential confounding factors, including long-term and seasonal trends, relative humidity, wind speed, mean temperature, sunshine duration, day of the week effect and holiday effect, were controlled in the following model:


$$Yt\sim \mathrm{quasi}\_\mathrm{Poisson}\;\left(\mu t\right)$$

$$\begin{array}{l} \begin{array}{l} L\mathrm{og}\left[E\left(Yt\right)\right]=\alpha +\beta EPt,l,3+ns\left(Time,7\right)+\\ ns\left(AT,3\right)+ns\left(RH,3\right)+ns\left(WS,3\right) \end{array}\\ +ns\left(SD,3\right)+\delta DOW+\gamma Holiday \end{array}$$

The symbols in this formula have the following meanings: *E* (Yt) is the expected number of AAD hospitalizations on day *t*; α and β represent the intercept and the cross-basis matrix coefficient, respectively; and ns () is the natural cubic spline function. The present study used the “ns-ns” method to construct the cross-basis of extreme precipitation (21), the degree of freedom (*df*) of the exposure dimension was set to 1, and the *df* of the lag dimension was set to 3. The effects were obtained as extreme precipitation relative to no precipitation. The model used a ns with 7 df/year to control the long-term trend and seasonality. SD, RH, AT and WS represent sunshine duration, relative humidity, average temperature and wind speed on day t with 3 df, respectively. Dow was used as a categorical variable to adjust the day of the week effect, and Holiday was used as a dummy variable to adjust the holiday effect.

According to previously published studies (22, 23) and the minimum quasi-Akaike information criteria (Supplementary Table A2), the lag period of extreme precipitation was set to 14 days. We also performed stratified analysis according to sex and age to identify populations that were sensitive to extreme precipitation.

To better reflect the AAD disease burden caused by extreme precipitation, the forwards perspective method was used to calculate the corresponding attributable fraction (AF) and number (AN) (25). The formulas used are shown below:


AFt=(RRt−1)/RRt

$$ANt=AFt^{*}Nt$$

RR is the maximum single-day lag relative risk value for this study, which was calculated by comparing extreme precipitation with no precipitation. *N_t_* is the number of AAD hospitalizations on day *t*. *AF_t_* represents the risk of hospitalizations for AAD due to extreme precipitation on day *t*.

Model fitting was performed in R (version 4.0.2) software using the “splines” and “dlnm” packages. Two-sided and *p* < 0.05 were considered statistically significant.

### Sensitivity analysis

We tested the robustness of the results by taking the following steps. First, the *df* of long-term and seasonal trends in the model was adjusted to 6–8. Second, the *df* of meteorological variables (sunshine duration, wind speed, relative humidity and mean temperature) was adjusted to 3–6. Third, studies have reported that air pollutants were correlated with an increased risk of cardiovascular disease (26–28). Therefore, we adjusted for PM_2.5_, SO_2_, and NO_2_ in the model. Finally, the precipitation boundary values (P90, P92.5, P97.5, and P99) were changed to observe the change in the results.

## Results

Tables 1, 2 display the basic description of daily AAD, weather conditions and pollutant concentrations in Shantou from 2015 to 2020. A total of 3,216 hospitalized cases of AAD were collected during the study period, with a maximum of 9 hospitalized cases per day. Most AAD patients were under 60 years old and accounted for 72.7% of the total number of AAD patients. The number of females admitted to the hospital with AAD was approximately 1.5 times that of males (1985 vs. 1,231). The daily rainfall, sunshine duration, atmospheric pressure, wind speed, relative humidity and mean temperature were 4.2 mm, 5.6 h, 1013.3 hPa, 3.6 m/s, 76.2% and 23.6°C, respectively.

**Table 1:** Characteristics of daily acute aortic dissection hospitalizations in Shantou, China, from 2015 to 2020.

Subgroups	Sum	Minimum	Maximum
Total	3,216	0	9
*Age groups*			
<60 years old	2,341	0	3
≥ 60 years old	875	0	9
*Gender groups*			
Female	1985	0	9
Male	1,231	0	3

**Table 2:** Descriptive statistics of weather conditions and pollutant concentrations from 2015 to 2020.

Variables	Mean (standard deviation)	Minimum	Frequency distribution				Maximum
			P10	P25	P75	P90	
Meteorological variables							
Mean temperature (°C)	23.6 (5.6)	5.1	15.7	18.8	28.6	30.3	33.2
Relative humidity (%)	76.2 (11.1)	36.5	61.3	69.0	84.3	90.0	100
Wind speed (m/s)	3.6 (0.9)	1.4	2.6	3.0	4.0	4.5	10.2
Sunshine duration (h)	5.6 (4.0)	0.0	0.0	1.0	9.0	10.5	12.3
Atmospheric pressure (hPa)	1013.3 (6.7)	993.8	1004.7	1008.1	1018.4	1021.9	1034.1
Rainfall (mm)	4.2 (12.8)	0.0	0.0	0.0	0.7	12.6	186.6
Air pollutants							
PM_2.5_ (μg/m^3^)	26.6 (14.0)	3	12	16	34	45	108
NO_2_ (μg/m^3^)	19.1 (8.1)	4	10	13	24	30	68
SO_2_ (μg/m^3^)	11.2 (4.1)	4	7	8	13	17	44

The daily average concentrations of SO_2_, PM_2.5_ and NO_2_ were 11.2 μg/m^3^, 26.6 μg/m^3^, and 19.1 μg/m^3^, respectively. During the study period, 110 days of extreme precipitation events (daily precipitation ≥26.25 mm) occurred. The time series distribution of weather factors is shown in Supplementary Figure A1, which indicates that the meteorological factors had obvious seasonal variation trends.

**Figure 2:**
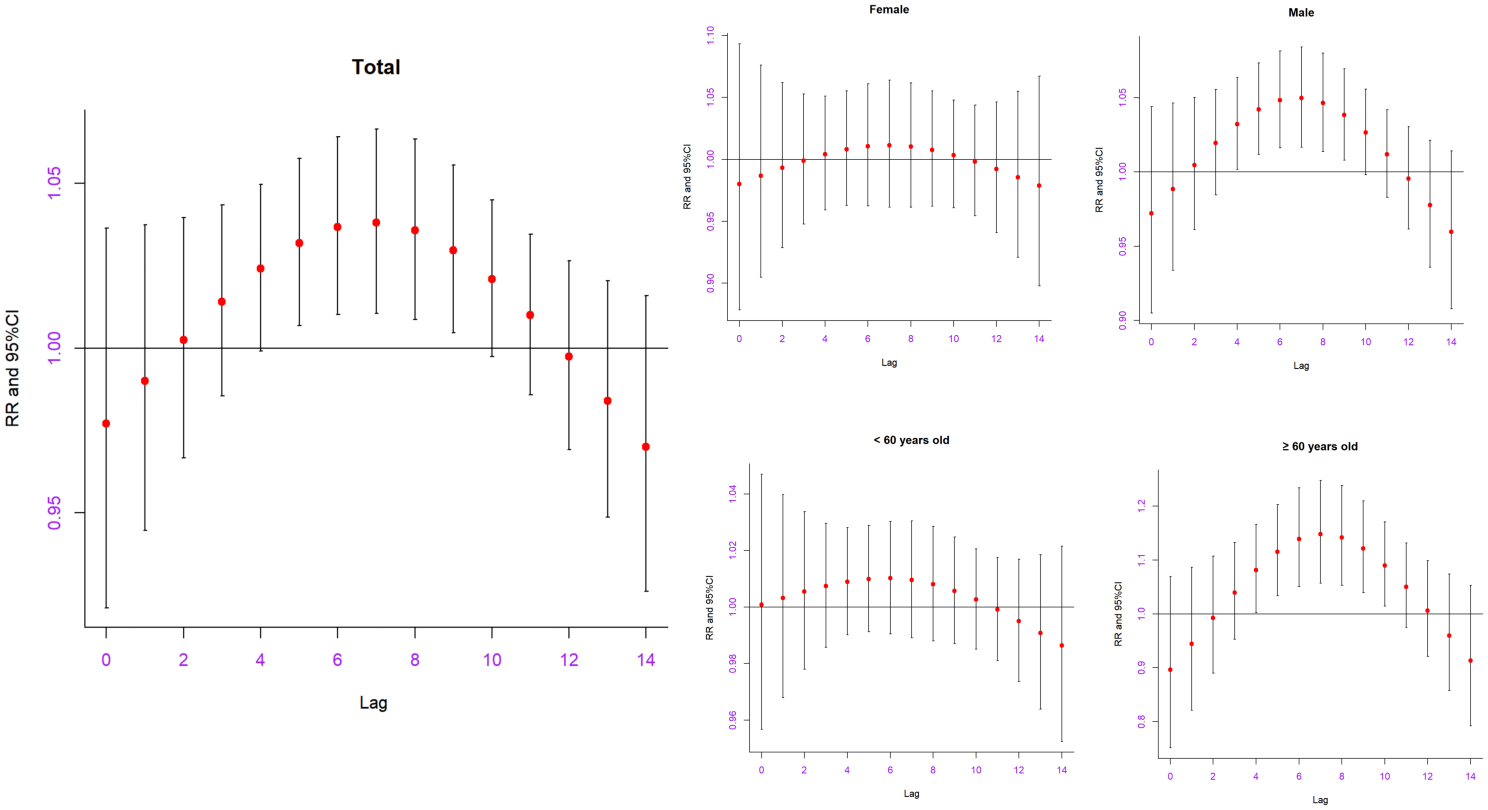
The relative risk of extreme precipitation on hospitalizations for AAD in the total and different subgroups on different lag days in Shantou, China.

Figure 2 shows the single-day lag effects of extreme precipitation on hospitalizations for AAD in Shantou, China. The significant adverse effect of extreme precipitation lasted from lag 5 (RR: 1.0318, 95% CI: 1.0067–1.0575) to lag 9 (RR: 1.0297, 95% CI: 1.0045–1.0555) and reached a maximum at lag 7 (RR: 1.0382, 95% CI: 1.0105–1.0665) (Table 3). The significant effects in the male group lasted from lag 4 (RR: 1.0321, 95% CI: 1.0017–1.635) to lag 9 (RR: 1.0382, 95% CI: 1.0079–1.0694) and reached its maximum at lag 7 (RR: 1.0498, 95% CI: 1.0165–1.841). The significant effects in the older adult individuals (≥60 years) lasted from lag 4 (RR: 1.0809, 95% CI: 1.0021–1.1660) to lag 10 (RR: 1.0897, 95% CI: 1.0145–1.1704) and reached its maximum at lag 7 (RR: 1.1481, 95% CI: 1.0566–1.2475). No significant effects of extreme precipitation were found in female and younger individuals (< 60 years). Supplementary Table A3 shows the cumulative lag effects on hospitalizations for AAD in Shantou, China. However, no significant effects of extreme precipitation were found in the total or subgroup AAD population.

**Table 3:** Effects of extreme precipitation on hospitalization for acute aortic dissection in Shantou, China.

Lag	Total	Female	Male	<60 years	≥ 60 years
0	0.9770 (0.9211–1.0363)	0.9800 (0.8784–1.0933)	0.9720 (0.9050–1.0440)	1.0008 (0.9567–1.0470)	0.8964 (0.7516–1.0690)
1	0.9899 (0.9446–1.0374)	0.9866 (0.9046–1.0760)	0.9885 (0.9338–1.0464)	1.0032 (0.9679–1.0398)	0.9442 (0.8207–1.0863)
2	1.0025 (0.9667–1.0396)	0.9930 (0.9286–1.0619)	1.0045 (0.9611–1.0500)	1.0054 (0.9779–1.0337)	0.9926 (0.8900–1.1069)
3	1.0141 (0.9855–1.0435)	0.9989 (0.9477–1.0529)	1.0194 (0.9845–1.0555)	1.0074 (0.9857–1.0296)	1.0391 (0.9530–1.1329)
4	1.0241 (0.9990–1.0497)	1.0040 (0.9591–1.0509)	1.0321 (1.0017–1.0635)^*^	1.0089 (0.9901–1.0281)	1.0809 (1.0021–1.1660)^*^
5	1.0318 (1.0067–1.0575)^*^	1.0079 (0.9628–1.0551)	1.0420 (1.0118–1.0732)^*^	1.0099 (0.9912–1.0289)	1.1152 (1.0338–1.2030)^*^
6	1.0367 (1.0101–1.0641)^*^	1.0105 (0.9624–1.0610)	1.0482 (1.0161–1.0813)^*^	1.0101 (0.9904–1.0303)	1.1386 (1.0508–1.2337)^*^
7	1.0382 (1.0105–1.0665)^*^	1.0113 (0.9613–1.0639)	1.0498 (1.0165–1.0841)^*^	1.0096 (0.9891–1.0305)	1.1481 (1.0566–1.2475)^*^
8	1.0356 (1.0086–1.0634)^*^	1.0103 (0.9614–1.0617)	1.0463 (1.0138–1.0798)^*^	1.0081 (0.9880–1.0285)	1.1417 (1.0527–1.2382)^*^
9	1.0297 (1.0045–1.0555)^*^	1.0076 (0.9620–1.0553)	1.0382 (1.0079–1.0694)^*^	1.0057 (0.9870–1.0248)	1.1212 (1.0393–1.2095)^*^
10	1.0209 (0.9974–1.0449)	1.0034 (0.9609–1.0478)	1.0265 (0.9981–1.0556)	1.0026 (0.9851–1.0205)	1.0897 (1.0145–1.1704)^*^
11	1.0099 (0.9858–1.0346)	0.9982 (0.9545–1.0438)	1.0119 (0.9829–1.0418)	0.9991 (0.9809–1.0175)	1.0503 (0.9751–1.1313)
12	0.9974 (0.9692–1.0264)	0.9921 (0.9406–1.0464)	0.9954 (0.9616–1.0303)	0.9950 (0.9736–1.0169)	1.0060 (0.9208–1.0990)
13	0.9839 (0.9486–1.0204)	0.9855 (0.9207–1.0549)	0.9777 (0.9358–1.0214)	0.9907 (0.9637–1.0185)	0.9595 (0.8575–1.0737)
14	0.9700 (0.9262–1.0159)	0.9787 (0.8977–1.0671)	0.9596 (0.9080–1.0141)	0.9864 (0.9524–1.0215)	0.9133 (0.7922–1.0531)

^*^p < 0.05.

Because no significant cumulative effects between extreme precipitation and AAD were found, the maximum single-day lag effect (at lag7) was selected to calculate the disease burden. Table 4 displays the AF and AN of AAD hospitalizations due to extreme precipitation. We found that the AF and AN of AAD hospitalizations were 3.68% (1.04, 6.24%) and 118 (33, 200), respectively. The AF and AN of male patients were 4.74% (1.62, 7.76%) and 58 58 (19, 95), respectively, which were both higher than those of female patients [AF: 1.12% (−4.03, 6.01%), AN: 21 (−80, 119)]. In the older adult group (≥ 60 years), 12.9% (113 cases) of AAD hospitalizations were due to extreme precipitation, and the corresponding AF and AN in the younger group (<60 years) were 0.95% and 22, respectively. These results indicated that extreme precipitation caused a higher disease burden in male and older adult individuals (≥60 years).

**Table 4:** Attributable fractions and number of AAD hospitalizations due to extreme precipitation in Shantou, China.

Group	AN	AF
Total	118 (33, 200)	3.68% (1.04, 6.24%)
Male	58 (19, 95)	4.74% (1.62, 7.76%)
Female	21 (−80, 119)	1.12% (−4.03, 6.01%)
<60 years old	22 (−25, 69)	0.95% (−1.10, 2.96%)
≥ 60 years old	113 (47, 174)	12.90% (5.36, 19.84%)

AAD acute aortic dissection, AN attributable number, AF attributable fraction.

After adjusting for the *df* of time (6–8) and meteorological factors (3–6) in the model, the association between extreme precipitation and AAD did not change significantly (Supplementary Figures A2–A6), which indicated that our results were robust. The estimated effects did not change significantly when pollutants were added to the model (PM_2.5_, NO_2_ and SO_2_) or different cut-off points for extreme precipitation were used (Supplementary Figures A7, A8).

## Discussion

With global climate change, extreme weather events have become more frequent and intense. The present study examined the impacts of extreme precipitation on AAD hospitalizations in Shantou, a coastal city in southern China, and found that the significant adverse effects lasted from lag 5 to lag 9 and reached a maximum at lag 7 (RR: 1.0382, 95% CI: 1.0105–1.0665). Subgroup results showed that males and older adult individuals (≥60 years) were more susceptible to extreme precipitation. The AF and AN of AAD hospitalizations were 3.68% and 118, respectively. Our study adds to the growing but limited evidence of extreme precipitation on acute cardiovascular events.

AAD is an acute critical cardiovascular disease (29). The incidence of AAD has continued to rise in recent decades, which has brought a heavy burden of disease to families and society (30). Studies have shown that targeted intervention measures for risk factors for AAD effectively reduce the morbidity and mortality of the disease (31). Therefore, it is necessary to investigate and identify risk factors associated with AAD. This study is the first to highlight the critical role of extreme precipitation in the progression of AAD.

Our results showed that extreme precipitation was significantly associated with AAD hospitalizations, which is consistent with two published studies on the relationship between extreme precipitation and cardiovascular disease (20, 22). Time series studies in Hefei (22) and Beijing (20) showed that extreme precipitation was significantly associated with ischaemic stroke and acute myocardial infarction admissions, respectively. However, AAD is a serious cardiovascular emergency with a serious prognosis, and it is the most common fatal disease involving the aorta (32). Many scholars have investigated the association between meteorological factors and AAD, but there are few studies involving rainfall, and the conclusions are inconsistent. A Beijing study examined the association between meteorological data and the morbidity of 345 patients with acute aortic dissection and found no significant association between daily average rainfall and AAD (RR: 1.00, 95% CI: 0.99–1.00, *p* = 0.07) (13). A study in subtropical Hong Kong used linear regression analysis to investigate the effects of meteorological factors on AAD or ruptured aortic aneurysm and found that rainfall was not associated with AAD or aortic aneurysm rupture (14). Another study in Urumqi, Xinjiang divided AAD into “days with AAD” and “days without AAD” and found no significant difference in daily rainfall (15). Two Japanese studies also reported that daily rainfall with AAD was not significantly different from rainfall without AAD (16, 33). However, some studies have shown that rainy days may be a risk factor for cardiovascular disease in the older adult (16, 22). The inconsistencies between the above conclusions may have been due to different study designs, study methods or study populations. More studies with different geographical characteristics are needed in the future to validate our conclusions.

The following mechanisms may explain extreme precipitation triggering of the onset of AAD. First, as an extreme weather event, extreme precipitation is often accompanied by significant changes in temperature, and low temperatures, diurnal temperature differences, and temperature variations are associated with the occurrence of AAD (34, 35). Second, the body’s perspiration capacity is reduced in a humid environment when extreme precipitation occurs, and the excretion of sodium and chloride ions is blocked, resulting in hypertension, which is the main trigger of AAD (36). Finally, most patients with AAD have comorbid cardiovascular diseases, and extreme precipitation may be an inducement to increase the possibility of AAD in patients with cardiovascular diseases.

Extreme precipitation caused a higher disease burden for males than females, and males seemed to be more susceptible to extreme precipitation. This difference may be because males work outdoors more often and have more direct exposure to extreme precipitation. More males in China smoke, which is associated with AAD (37, 38). Older adult people (≥ 60 years) were more vulnerable to extreme precipitation than younger people (<60 years). This result is consistent with the results of a Japanese study in which the daily precipitation of older adult patients with aortic dissection was significantly higher than that of young patients (16). This difference may be due to the weak body resistance of older adult individuals, who are unable to tolerate the impact of adverse environmental changes (22). Older adult people are more likely to have underlying medical conditions, such as heart disease and hypertension, and more likely to develop AAD under the adverse effects of extreme precipitation (39).

The present study has several advantages. First, as an acute and critical cardiovascular disease, most patients with AAD choose general hospitals with authority and a high medical level. The present study included four top-level general hospitals in Shantou, which basically covered the hospitalized population of AAD patients in Shantou and increased the reliability of the results. Second, to the best of our knowledge, this study may be the first study to investigate the impacts of extreme precipitation on AAD hospitalizations in a coastal city of southeast China using DLNM. Third, previous studies reported that air pollution was correlated with AAD admissions (6, 7). Therefore, we also adjusted for PM_2.5_, SO_2_, and NO_2_ in the sensitivity analysis. Finally, we investigated the association between extreme precipitation and AAD and the lag effects of AAD, which identified the vulnerable population of extreme precipitation and calculated the attributable disease burden. The present study provides a basis for people to better understand the harm of extreme precipitation on AAD and apply more targeted interventions.

Several limitations should not be ignored. First, the present study set extreme precipitation as a categorical variable, which is a method that is used more often in extreme precipitation-related studies (20, 22), and more accurate classification and analysis methods for extreme precipitation are needed in the future. Second, our study was performed only in a coastal city in southeast China, Shantou, and the results may not be applicable to other regions with different climatic characteristics. Third, confounding factors, such as smoking, diet, disease history and socioeconomic status, may also influence AAD hospitalizations (40–42), but these data were not available. Fourth, the environmental data came from fixed monitoring stations, which leads to unavoidable exposure measurement errors. Fifth, this study is an ecological study and cannot explain the causal association between extreme precipitation and AAD. Finally, due to limitations in data sources, we were unable to stratify AAD (e.g., types A and B), and different disease subtypes may differ in sensitivity to extreme precipitation, which will be considered in future studies.

## Conclusion

Our results showed that extreme precipitation was significantly associated with AAD hospitalizations. Males and older adult individuals had a higher disease burden and were more susceptible to extreme precipitation. During the study period, 3.68% of AAD hospitalizations in Shantou were due to extreme precipitation. Our study supplements the current limited evidence of the relationship between extreme precipitation and AAD and provides a reference for the relevant governments and medical departments to implement targeted extreme precipitation intervention and control measures.

## Data availability statement

The data analyzed in this study is subject to the following licenses/restrictions: the data that support the findings of this study are available from the corresponding author, upon reasonable request. Requests to access these datasets should be directed to lpli@stu.edu.cn.

## Ethics statement

Prior to data collection, the Ethics Committee of Shantou University approved this study. Overall, aggregated data were used in our study, and no information about individual patient privacy was involved in the analysis.

## Author contributions

YJ and LL: conceptualization, data curation, investigation, and writing–original draft. JX and ZY: validation and formal analysis. ZY and ZH: conceptualization and writing–review and editing. YJ and JX: writing–review and editing and supervision. All authors contributed to the article and approved the submitted version.

## Conflict of interest

The authors declare that the research was conducted in the absence of any commercial or financial relationships that could be construed as a potential conflict of interest.

## Publisher’s note

All claims expressed in this article are solely those of the authors and do not necessarily represent those of their affiliated organizations, or those of the publisher, the editors and the reviewers. Any product that may be evaluated in this article, or claim that may be made by its manufacturer, is not guaranteed or endorsed by the publisher.

## Footnotes

^1^ http://data.cma.cn/
