# Supplementary material for: Risk assessment and disease burden of extreme precipitation on hospitalizations for acute aortic dissection in a subtropical coastal Chinese city
Source: Front Public Health. 2023 Jun 29;11:1216847. doi: 10.3389/fpubh.2023.1216847 (PMC10343949; doi:10.3389/fpubh.2023.1216847)
Supplement: Supplementary file 1 [file Table_1.DOCX]

**Risk assessment and disease burden of extreme precipitation on hospitalizations for acute aortic dissection in a subtropical coastal Chinese city**

Yanhu Ji^1^, Jianping Xiong^2^, Zhongjia Yuan^3^, Zepeng Huang^4^, Liping Li^1,5^**^*^**

**^*^ Corresponding author:** Liping Li

E-mail: lpli@stu.edu.cn

**Table A1** Spearman correlation coefficients between air pollutants and weather factors in Shantou

|  | PM_2.5_ | SO_2_ | NO_2_ | Rainfall | Atmospheric pressure | Mean temperature | Relative humidity | Sunshine duration |
| --- | --- | --- | --- | --- | --- | --- | --- | --- |
| SO_2_ | 0.63^***^ | 1 |  |  |  |  |  |  |
| NO_2_ | 0.67^***^ | 0.63^***^ | 1 |  |  |  |  |  |
| Rainfall | -0.30^***^ | -0.24^***^ | -0.061^**^ | 1 |  |  |  |  |
| Atmospheric pressure | 0.4^***^ | 0.20^***^ | 0.55^***^ | -0.27^***^ | 1 |  |  |  |
| Mean temperature | -0.39^***^ | -0.14^***^ | -0.61^***^ | 0.019 | -0.86^***^ | 1 |  |  |
| Relative humidity | -0.23^***^ | -0.28^***^ | -0.089^***^ | 0.59^***^ | -0.32^***^ | 0.088^***^ | 1 |  |
| Sunshine duration | -0.025 | 0.085^***^ | -0.27^***^ | -0.49^***^ | -0.19^***^ | 0.42^***^ | -0.42^***^ | 1 |
| Wind speed | -0.28^***^ | -0.25^***^ | -0.41^***^ | 0.037 | -0.28^***^ | 0.29^***^ | -0.065^**^ | 0.19^***^ |

**Note:** ^*^*P* < 0.05; ^**^*P* < 0.01; ^***^*P* < 0.001.

**Table A2** The AIC values from lag 1 to lag 14.

| Lag | Q-AIC | Lag | Q-AIC |
| --- | --- | --- | --- |
| 1 | 5890.576 | 8 | 5871.505 |
| 2 | 5887.875 | 9 | 5870.031 |
| 3 | 5885.55 | 10 | 5869.38 |
| 4 | 5884.246 | 11 | 5867.578 |
| 5 | 5881.223 | 12 | 5865.561 |
| 6 | 5877.257 | 13 | 5863.390 |
| 7 | 5875.036 | 14 | 5860.736 |

**Table A3**The cumulative lag effects on hospitalization for acute aortic dissection among the total sample and different subgroups in Shantou, China

| Lag | Total | Male | Female | <60 years old | ≥ 60 years old |
| --- | --- | --- | --- | --- | --- |
| 0-0 | 0.9770 (0.9211-1.0363) | 0.9720(0.9050-1.0440) | 0.9800(0.8784-1.0933) | 1.0008(0.9567-1.0470) | 0.8964(0.7516-1.0691) |
| 0-1 | 0.9672(0.8703-1.0749) | 0.9609(0.8453-1.0922) | 0.9669(0.7950-1.1761) | 1.0041(0.9261-1.0885) | 0.8464(0.6173-1.1605) |
| 0-2 | 0.9696( 0.8422-1.1164) | 0.9653(0.8134-1.1455) | 0.9602(0.7397-1.2465) | 1.0095(0.9064-1.1244) | 0.8402(0.5512-1.2806) |
| 0-3 | 0.9833 (0.8324-1.1615) | 0.9840(0.8036-1.2049) | 0.9592(0.7050-1.3050) | 1.0170(0.8954-1.1551) | 0.8730(0.5303-1.4373) |
| 0-4 | 1.0070 (0.8374-1.2110) | 1.0157(0.8114-1.2714) | 0.9630(0.6852-1.3535) | 1.0261(0.8913-1.1814) | 0.9437(0.5428-1.6407) |
| 0-5 | 1.0391 (0.8532-1.2654) | 1.0585(0.8326-1.3455) | 0.9707(0.6752-1.3957) | 1.0363(0.8916-1.2045) | 1.0525(0.5822-1.9028) |
| 0-6 | 1.0772 (0.8761-1.3246) | 1.1095(0.8626-1.4272) | 0.9810(0.6704-1.4354) | 1.0469(0.8942-1.2257) | 1.1984(0.6427-2.2347) |
| 0-7 | 1.1184 (0.9015-1.3873) | 1.1648(0.8961-1.5141) | 0.9921(0.6670-1.4756) | 1.0570(0.8969-1.2456) | 1.3760(0.7171-2.6401) |
| 0-8 | 1.1583 (0.9253-1.4499) | 1.2188(0.9277-1.6012) | 1.0024(0.6625-1.5167) | 1.0655(0.8981-1.2642) | 1.5710(0.7952-3.1036) |
| 0-9 | 1.1927 (0.9439-1.5072) | 1.2654(0.9526-1.6809) | 1.0100(0.6555-1.5563) | 1.0717(0.8968-1.2805) | 1.7615(0.8652-3.5860) |
| 0-10 | 1.2177 (0.9544-1.5536) | 1.2990(0.9669-1.7450) | 1.0135(0.6455-1.5912) | 1.0745(0.8927-1.2933) | 1.9195(0.9152-4.0260) |
| 0-11 | 1.2298 (0.9546-1.5845) | 1.3145(0.9672-1.7864) | 1.0117(0.6322-1.6190) | 1.0735(0.8853-1.3018) | 2.0161(0.9332-4.3554) |
| 0-12 | 1.2267 (0.9419-1.5975) | 1.3084(0.9505-1.8011) | 1.0038(0.6142-1.6404) | 1.0682(0.8737-1.3061) | 2.0282(0.9094-4.5235) |
| 0-13 | 1.2069 (0.9142-1.5934) | 1.2793(0.9142-1.7901) | 0.9893(0.5894-1.6603) | 1.0584(0.8566-1.3077) | 1.9463(0.8381-4.5194) |
| 0-14 | 1.1708 (0.8695-1.5764) | 1.2276(0.8569-1.7586) | 0.9683(0.5556-1.6875) | 1.0440(0.8324-1.3094) | 1.7778(0.7223-4.3752) |

**Note:** ^*^*P* < 0.05.
