# Supplementary figures and images for: Risk assessment and disease burden of extreme precipitation on hospitalizations for acute aortic dissection in a subtropical coastal Chinese city
Source: Front Public Health. 2023 Jun 29;11:1216847. doi: 10.3389/fpubh.2023.1216847 (PMC10343949; doi:10.3389/fpubh.2023.1216847)

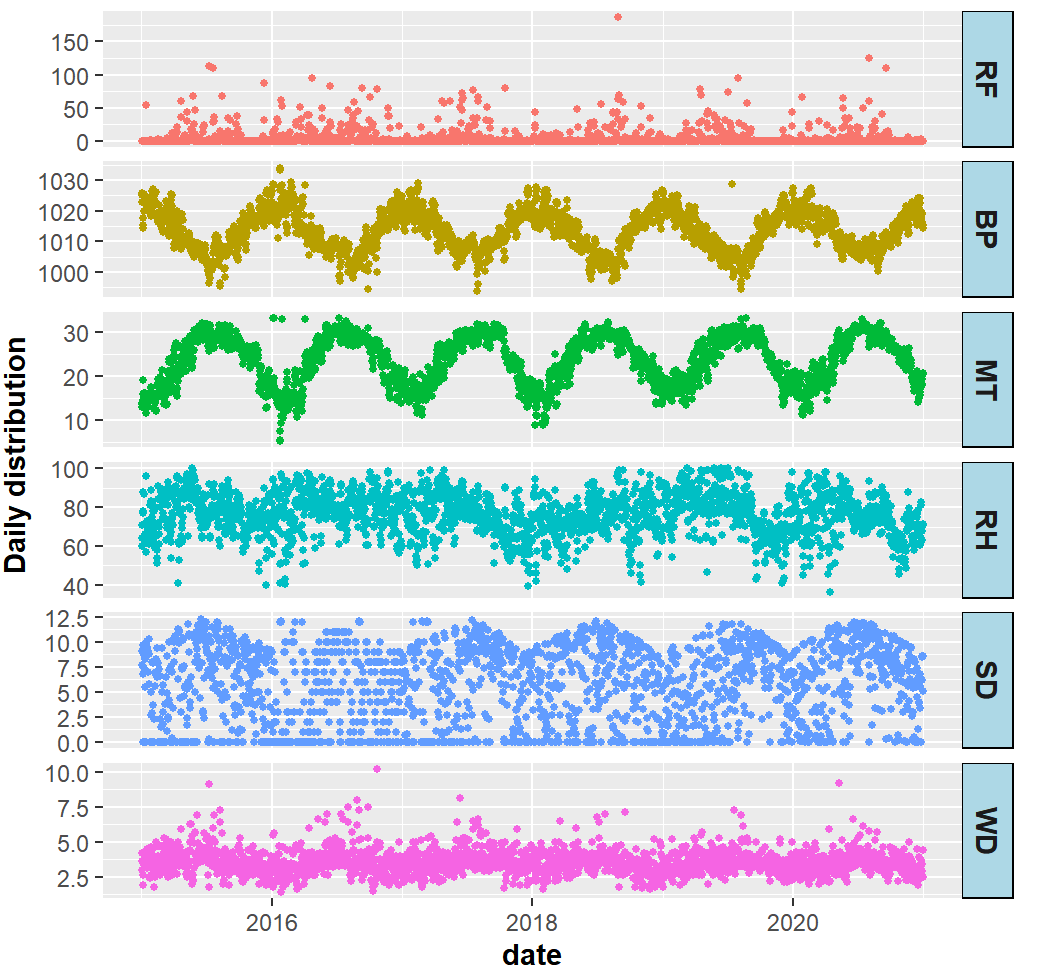

Supplement: Supplementary file 2 [file Image_1.TIF]
